# Give credit where credit is due, also for omics data

**DOI:** 10.1038/s44319-025-00680-6

**Published:** 2026-01-03

**Authors:** Ronald P de Vries, Mao Peng

**Affiliations:** Fungal Physiology, Westerdijk Fungal Biodiversity Institute, Uppsalalaan 8, 3584 CT Utrecht, The Netherlands

**Keywords:** Careers, Chromatin, Transcription & Genomics, Science Policy & Publishing

## Abstract

The exponentially increasing amount of omics data has created problems regarding ethical use of data generated by others. We address some of these issues and make suggestions on how they could be avoided or solved to ensure an open and fair research environment.

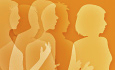

The rapidly increasing amount of omics data being generated has sparked a major change in biological research and has provided unprecedented insights into biological processes and diversity. Much of the data is made publicly available, sometimes even before the primary paper describing the data is published, which enables easy access and re-analysis. This sharing and reuse of biological data have been widely and strongly encouraged (Wilson et al, 2021) but ensuring that the people who generated the data in the first place are properly acknowledged is still lacking. While there are systems in place to ensure this (Hug et al, 2025; Oza et al, 2023), they are regularly ignored, misunderstood or not enforced. This may—and already does—result in scientists delaying providing public access to their data, to avoid being scooped on the data they themselves generated. We would therefore like to draw attention to and spark discussion about three common issues regarding data citation: not citing published omics data, unauthorized use of unpublished data and not making published data publicly available. Our experience is mainly related to fungal biology and biotechnology, but it is clear that these issues occur in all fields of biology.

The large amount of omics data publicly available lends itself to an increasing number of comparative studies, which often include data from multiple papers—and often do not cite the original papers, but rather the repository from which they obtained the data. While we agree that these repositories should be credited for facilitating use of omics data, this does not remove the rights of the people who generated the data.

Students are taught to cite a paper they obtain data from—whether this is a gene sequence, biochemical data on an enzyme, image, software and code or anything else. This should also apply for omics data: the original papers describing the data that has been reused should be cited. In discussions about this, people usually comment that this is not always possible as journals often put limits on the number of citations in a new manuscript. This is indeed the case, but many referencing platforms are now able to extract citations from other sources than just the reference list in a paper. A relatively simple solution to solve this problem is to provide a supplemental table with the details on the data used in a manuscript, including the original citations. These are then not part of the reference list but can still be sourced by referencing platforms thereby ensuring credits for the original authors and data creators. This is particularly relevant for PhD students and young postdocs, who typically not have an extensive publication list and citations to their papers are crucial for their career prospects. Giving due credit to data creators will also foster data sharing, progress and potential collaboration. If we only cite the repositories, we place the burden of identifying the origin of the data on the reader, while this should be clearly the task of the authors.

Several repositories and websites that host omics data already provide public access to the data before the original paper is published. The main example in the fungal biology field is the Mycocosm Portal from the Joint Genome Institute (USA) (Grigoriev et al, 2014). It contains many fungal genomes sequences which are publicly accessible, but not (yet) published. The data policy describes how to use such data, depending on when it was generated and made public, typically requiring to ask for permission from the PI whose group generated the data. These policies are clearly stated on the portal but they are not enforced. Unfortunately, and not too surprising, manuscripts are regularly submitted in which these policies have not been followed, thereby compromising the right of the people who generated the data and sometimes even scooping them on their own data. It does not appear that these aspects are rigorously checked during the reviewing process of a manuscript, which is probably the only moment this could be done.

We therefore suggest that scientific journals enforce a stricter data policy that requires authors to provide a table with the data that was used and whether this data has already been published or not. If it was published, the citation should be included and not just the accession number of a repository. If it is unpublished data, the people that generated the data should be mentioned and that the authors of the current manuscript have permission to use this data. Even for publicly available data without corresponding publications, such as many genomes and transcriptomes in NCBI genome and GEO databases, it is recommended to acknowledge the data creator. These measures would allow a much easier evaluation of correct data use and citations for reviewers and will also stimulate authors to correctly credit the people who generated it. More importantly, it will create a safe, friendly and trusted environment for all parties, including data creator, data distributors and data consumers.

Finally, we feel that a simpler policy should be in place to access published data. Currently, most journals require that data from used in a manuscript is made publicly available, but the policies differ between journals and publishers and are often not enforced. As a result, many papers based on, for instance, transcriptomic data do not mention where the data is deposited or state that ‘data is available upon request’. When we asked authors for the data, we have obtained it in less than 50% of the cases and often did not even receive a response. In addition, if repository numbers are included, the data in the repository is often classified as private and therefore not accessible.

It would help if journals also mandated authors to deposit their data in a public repository and require that it is publicly accessible at the moment of publication. This would require an additional editorial check, but it would ensure that other researchers could repeat and validate the study. More importantly, this data would be available for reuse to stimulate future studies and advance our overall understanding of biology.

The scientific community can only function effectively if we behave ethically, acknowledge the efforts of our colleagues and respect their rights. While we are well aware that views on ethics and integrity differ, the citing of scientists that generated the data you use in your study is a basic principle that we should all agree on. The sharing of omics data has greatly advanced biological research, but also caused practical problems for implementing equitable reuse of data. However, this should not be a reason to ignore other researchers’ efforts but rather think about how to minimize the tension between data creators and data consumers. The suggestions we made in this opinion paper are just some options, which we hope will increase awareness and discussion.

**Editorial note**: EMBO Press journals address these issues by allowing unlimited citations for reviews and research papers while encouraging citation of original publication; offering a specific data reference style; and mandating that all data used in a manuscript are deposited and made publicly available at the day of publication.

## Supplementary information

Peer Review File
