# Supplementary material for: Give credit where credit is due, also for omics data
Source: EMBO Rep. 2026 Jan 3;27(2):257–8. doi: 10.1038/s44319-025-00680-6 (PMC12852817; doi:10.1038/s44319-025-00680-6)
Supplement: Supplementary file 1 — Peer Review File [file 44319_2025_680_MOESM1_ESM.pdf]

EMBO reports

Give credit where credit is due, also for omics data

Ronald de Vries and Mao Peng

Corresponding author(s): Ronald de Vries (r.devries@wi.knaw.nl)

Review Timeline:	Submission Date:	8th Dec 25
	Accepted:	15th Dec 25

Editor: Holger Breithaupt

Transaction Report: This article type is not peer reviewed.
